# Histone Modifications and Chondrocyte Fate: Regulation and Therapeutic Implications

**DOI:** 10.3389/fcell.2021.626708

**Published:** 2021-04-16

**Authors:** Chao Wan, Fengjie Zhang, Hanyu Yao, Haitao Li, Rocky S. Tuan

**Affiliations:** ^1^MOE Key Laboratory for Regenerative Medicine, School of Biomedical Sciences, Faculty of Medicine, The Chinese University of Hong Kong, Hong Kong, China; ^2^Institute for Tissue Engineering and Regenerative Medicine, The Chinese University of Hong Kong, Hong Kong, China; ^3^MOE Key Laboratory for Regenerative Medicine (Shenzhen Base), School of Biomedical Sciences Core Laboratory, Shenzhen Research Institute, The Chinese University of Hong Kong, Shenzhen, China; ^4^MOE Key Laboratory of Protein Sciences, Beijing Advanced Innovation Center for Structural Biology, Beijing Frontier Research Center for Biological Structure, Tsinghua-Peking Joint Center for Life Sciences, Department of Basic Medical Sciences, School of Medicine, Tsinghua University, Beijing, China

**Keywords:** epigenetics, histone modification, chondrocyte, articular cartilage, regeneration, osteoarthritis

## Abstract

The involvement of histone modifications in cartilage development, pathology and regeneration is becoming increasingly evident. Understanding the molecular mechanisms and consequences of histone modification enzymes in cartilage development, homeostasis and pathology provides fundamental and precise perspectives to interpret the biological behavior of chondrocytes during skeletal development and the pathogenesis of various cartilage related diseases. Candidate molecules or drugs that target histone modifying proteins have shown promising therapeutic potential in the treatment of cartilage lesions associated with joint degeneration and other chondropathies. In this review, we summarized the advances in the understanding of histone modifications in the regulation of chondrocyte fate, cartilage development and pathology, particularly the molecular writers, erasers and readers involved. In addition, we have highlighted recent studies on the use of small molecules and drugs to manipulate histone signals to regulate chondrocyte functions or treat cartilage lesions, in particular osteoarthritis (OA), and discussed their potential therapeutic benefits and limitations in preventing articular cartilage degeneration or promoting its repair or regeneration.

## Introduction

During development, chondrogenesis is the process of cartilage formation from the condensing mesenchyme, which is tightly controlled by a number of growth factors including transforming growth factor-βs (TGF-βs), bone morphogenetic proteins (BMPs), fibroblast growth factors (FGFs), parathyroid hormone-related protein (PTHRP), Indian hedgehog (IHH) and Wnts, hormones such as growth hormone (GH), insulin like growth factor-1 (IGF-1), insulin, estrogen and androgen, as well as transcription factors including Sry-related HMG Box-9 (SOX9) and Runt-related transcription factor 2 (RUNX2), etc. Those local and systemic regulators work with key transcription factors to form a complex regulatory network to control the development, growth and homeostasis of the skeleton (Goldring et al., 2006; Baron et al., 2015; Carballo et al., 2017). There are two functional locations for cartilage in the skeleton: the growth plate (or epiphyseal plate) and articular cartilage, representing transitional cartilage and permanent cartilage, respectively. The growth plate is the place where bone forms via an endochondral ossification process that defines the longitudinal growth of long bones. Defects in the development of cartilage leads to various, growth disorders, known as osteochondrodysplasia. A most common defect is achondroplasia that causes dwarfism or short stature and deformities of bones and joints (Ornitz and Legeai-Mallet, 2017). Articular cartilage is an avascular, aneural, resilient connective tissue covering and protecting the ends of long bones of joints. It has very limited capacity of repair or regeneration following degeneration or injury. The volume, quantity, structure and organization of the extracellular matrix (ECM) components of articular cartilage, including collagen fibers (e.g., collagen types II and IX), proteoglycans and glycosaminoglycans (GAGs), are the primary determinants of normal joint function. This complex composition is regulated by chondrocytes in response to the changes in their chemical and mechanical environment (Prein and Beier, 2019). Degeneration of articular cartilage, resulting from dysfunction of chondrocytes or abnormal degradation of its ECM components, is a key feature of OA and associated with failure of articular cartilage repair. Due to the relatively poor understanding of the etiology and pathogenesis, delayed diagnosis, lack of defined drug targets, and inefficient drug delivery approaches, the treatment of these chondropathies remains a significant clinical challenge.

Among the various chondropathies, OA is the most prevalent joint disease causing severe pain and significant economic cost to patients and the society. The pathology of OA is characterized by progressive destruction, thinning or wearing-out of articular cartilage, resulting in pain and limited movement of the joint. The genetics of OA is complex, as it does not usually follow the typical pattern of Mendelian inheritance. Prevailing evidence supports the theory of a polygenic inheritance but not a single gene mutation for the genetic basis of OA. Genome-wide association studies reveal that a panel of loci and candidate genes are associated with the pathogenesis of OA (Lawrence, 2016). Much attention has been focused on the interaction between genetic and environmental factors (e.g., obesity, mechanical stress, and hypoxia) that are critical for the initiation and progression of OA (Felson and Zhang, 1998). In fact, epigenetic modifications also play important roles in the pathogenesis of OA and other chondropathies (Fernández-Moreno et al., 2008; Coutinho de Almeida et al., 2017; Peffers et al., 2018).

Epigenetic modifications are the molecular events altering chromatin, gene expression and heritable phenotypes without changing the DNA sequence. The interactions between genes and transcriptional factors and modulators are usually modulated by DNA methylation, histone modifications, microRNAs and chromatin remodeling (van Wijnen and Westendorf, 2019). Epigenetic modifications play important roles in determining the fate of stem cells and chondrogenic lineage differentiation (Hata, 2015). Over the past few years, significant progress has been made in determining the role of epigenetics in chondrocyte fate decision and in the pathogenesis of multiple chondropathies. Several excellent reviews have highlighted the functions of DNA methylation, histone modifications, and non-coding RNAs in regulating the biological behavior of chondrocytes (Furumatsu and Ozaki, 2010; Barter et al., 2012; Duan et al., 2015; Hata, 2015; Kim et al., 2015; Bhonde et al., 2016; Ramos and Meulenbelt, 2017; Haq et al., 2018; Allas et al., 2019; Ren et al., 2020; Yang et al., 2020).

The family of histone proteins includes H2A, H2B, H3, H4, and multiple variants, each having unique functions. Histone proteins form an octamer around which DNA of ∼146-bp is wrapped to form a nucleosome, the basic chromatin unit. The nucleosomes assemble to form highly condensed chromatin, while individual genes can be accessed through localized loosening through post-translational modifications (PTMs) of the core histone proteins (Kouzarides, 2007). PTMs mainly include phosphorylation, acetylation, methylation, ubiquitination, SUMOylation, and GlcNAcylation by reversible incorporation of phosphate, acetyl groups, methyl groups, SUMO (small ubiquitin-like modifier), and *O*-GlcNAc (*O*-linked β-*N*-acetylglucosamine) within histone tails, respectively. The enzymes that add modifications are referred to as writers, which include histone acetyltransferases (HATs) and histone lysine methyltransferases (KMTs), whereas those that remove PTMs are called erasers, including histone deacetylases (HDACs) and histone lysine demethylases (KDMs). The readers refer to effector proteins that specifically recognize and bind histone marks. Multiple excellent reviews have highlighted the molecular regulatory mechanisms of these histone-modifying proteins (Ruthenburg et al., 2007; Yun et al., 2011; Wang et al., 2015; Zhang T. et al., 2015; Andrews et al., 2016; Patel, 2016; Hyun et al., 2017; Zhao et al., 2019).

**FIGURE 1:**
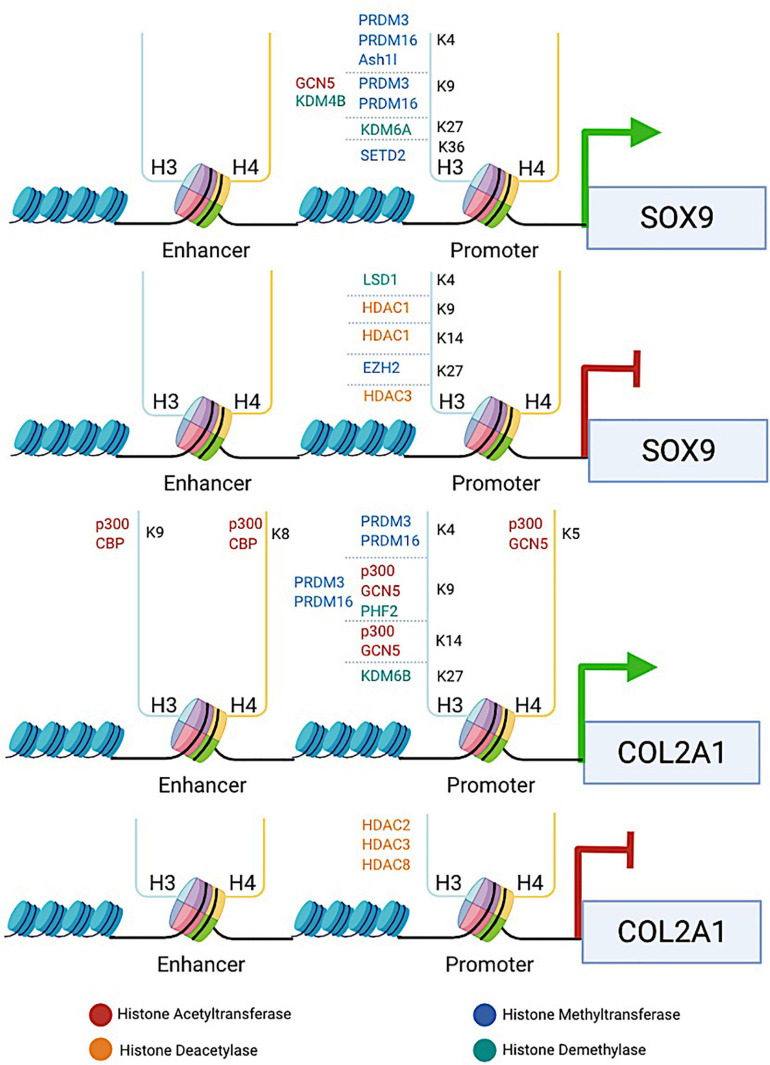
The diagram illustrates the involvement of histone-modifying proteins in enhancing or repressing the transcription of two key chondrogenic marker genes *SOX9* and *COL2A1*. HATs (e.g., p300, CBP, and GCN5), HDACs (e.g., HDAC1/2/3/8), HMTs (e.g., PRDM3/16, SETD2, and EZH2), and KDMs (e.g., KDM4B, KDM6A/6B, LSD1, and PHF2) are recruited to the specific lysine residue in the histone tail binding at the promoters or enhancers of *SOX9* and *COL2A1* genes. HATs induce histone hyperacetylation, which is associated with transcriptional activation of *SOX9* and *COL2A1*, whereas HDACs induce histone deacetylation, leading to transcriptional repression of both genes. HMTs and KDMs are involved in both transcriptional activation and repression of *SOX9* and *COL2A1*, depending on the site at which the particular enzyme catalyzes methylation. The promotion or repression of *SOX9* and *COL2A1* transcription by histone-modifying proteins provides an example to reveal how histone modifications are involved in regulating chondrocyte fate and ECM production.

**FIGURE 2:**
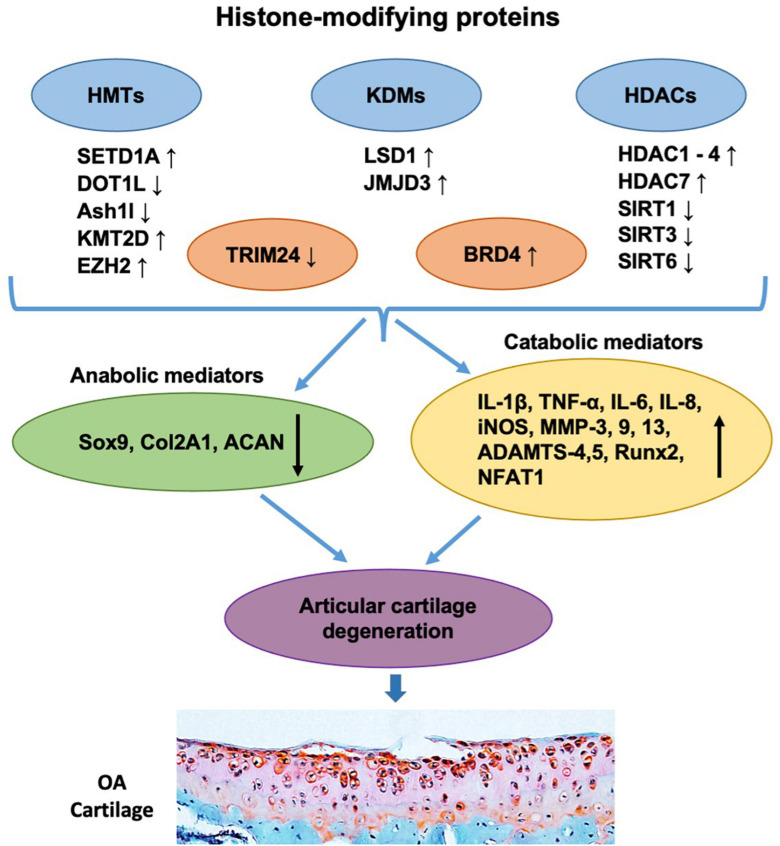
Histone-modifying proteins are involved in the pathogenesis and progression of OA. The levels of histone-modifying proteins including HMTs (e.g., SETD1A, DOT1L, Ash1l, KMT2D, and EZH2), KDMs (e.g., LSD1 and JMJD3), HDACs (e.g., HDAC1, HDAC2, HDAC3, HDAC4, HDAC7, SIRT1, SIRT3, and SIRT6), and readers (e.g., TRIM24 and BRD4) are shown to be changed in OA articular chondrocytes. These alterations further downregulate chondrocyte anabolic mediators (e.g., Sox9, Col2A1, and ACAN) and/or upregulate catabolic mediators (e.g., IL-1β, TNF-α, IL-6, IL-8, iNOS, MMP-3, 9, 13, ADAMTS-4,5, Runx2, and NFAT1), leading to the imbalance of chondrocyte metabolism, which subsequently causes articular cartilage degeneration and OA progression.

Prevailing evidence indicates that the histone-modifying proteins that regulate chondrocyte anabolic or catabolic gene expression programs in cartilage are instrumental in determining chondrocyte fate and functions (Tables 1–3, and Figures 1, 2). Histone marks are associated with transcriptional activity. Changes in histone marks, such as methylation levels of histone 3 lysine 4 trimethylation (H3K4me3) and H3K36me3, correlate with the alterations of gene expression levels from human mesenchymal stem cells (MSCs) to differentiated chondrocytes (Herlofsen et al., 2013). H3K4me3 and histone 3 lysine 27 acetylation (H3K27ac) are enriched in highly expressed genes, while H3K27me3 shows a greater enrichment in genes with low expression levels in different developmental stages of chondrocyte. Histone modifications around the enhancer and promoter of the key chondrocyte marker genes *SOX9* and collagen type II (*COL2A1*) switch from repression in human MSCs to transcriptionally active in differentiated chondrocytes (Wang et al., 2012; Nishimura et al., 2013; Cheung et al., 2020). Therefore, a comprehensive understanding of the histone modifications associated with cartilage development and pathology is of great significance for understanding the precise control of chondrocyte fate and discovery of novel therapeutic approaches for multiple chondropathies. In this review, we particularly focus on the progress of the molecular writers, erasers and readers of histone modifications in controlling the fate and functions of chondrocytes under pathophysiological conditions, and discuss the potential therapeutic benefits and limitations of targeting histone modifications for the treatment of OA or promoting articular cartilage repair or regeneration.

**TABLE 1:** Histone-modifying enzymes serve as writers to regulate chondrocyte fate.

	Enzyme	Histone	Genes regulated	Species	Effects on chondrocyte function	References
HAT	p300/CBP	H3K9, H4K8	*COL2A1*	Human	Promoting expression of *COL2A1*	Furumatsu et al., 2005
			*COMP*	Murine	Promoting expression of *COMP*	Liu et al., 2007
	p300/GCN5	H3K9/K14, H4K5	*COL2A1*	Human	Promoting expression of *COL2A1*	Dvir-Ginzberg et al., 2008
	GCN5	None-histone acetyltransferase	mTORCI	Murine	Inhibiting GCN5 disrupts the mTORC1 pathway during chondrocyte maturation	Pezoa et al., 2020
	GCN5 (KAT2A)	H3K9	*Sox9, Col2A1*	Zebrafish	Promoting expression of *Sox9* and *Col2A1*	Sen et al., 2018
HMT	SETDB1 (ESET)	H3K9	*Osteocalcin*	Murine	Repressing transcription of *osteocalcin*	Yang et al., 2013
			*Alp, Mmp13*	Murine	Inhibiting chondrocyte hypertrophy	Lawson et al., 2013b
			*Sox9, Acan, Col2A1, Mmp13*	Murine	Promoting expression of *Sox9, Acan, Col2A1, Mmp13*	Yahiro et al., 2017
	GLP (Ehmt 1)	H3K9me2	*Runx2*	Murine	Repressing transcription of *Runx2*	Balemans et al., 2014
	PRDM3, PRDM16	H3K4me3, H3K9me3	*Col2A1*, Sox9	Zebrafish murine	PRDM3 promotes the expression of *Col2A1* and *Sox9*, loss of PRDM3/16 leads to craniofacial defects	Shull et al., 2020
	SUV39H1	H3K9me3	*miR-15a*	Human	Repressing miR-15a, increasing chondrocyte proliferation, upregulating *COL2A1* and *BCL2*	Li et al., 2019
	DOT1L	?	*COL2A1, COL10A1, ACAN*	Human	Promoting expression of *COL2A, COL10A1, ACAN*	Castaño Betancourt et al., 2012
		H3K79me2	*LEF1, TCF1*	Human	Repressing *LEF1* and *TCF1*, preventing Wnt hyperactivation	Monteagudo et al., 2017
	Ash1I	H3K4me3	*Runx2, Hoxa10, Sox9*	Murine	Promoting expression of *Runx2, Hoxa10, Sox9*	Yin et al., 2019
		H3K36me2	*Hox*	Murine	Promoting expression of Hox genes	Miyazaki et al., 2013
	KMT2D	H3K4me3	*Shox2*	Murine	Promoting expression of *Shox2*, inhibiting SOX9 activity	Fahrner et al., 2019
	SETD1A	H3K4me2/3	*iNOS, COX-2*	Human	Promoting expression of *iNOS, COX-2*	El Mansouri et al., 2011
	SETD2	H3K36me2/3	*BMP2, SOX9*	Human	Promoting expression of *BMP2*, SOX9	Fang et al., 2016
	EZH2	H3K27me3	*BMP2, SP7, IBSP, BGLAP*	Murine	Repressing *BMP2, SP7, IBSP, BGLAP*	Camilleri et al., 2018
	EZH1 and EZH2	?	*Ink4a/b*	Murine	Decreasing chondrocyte proliferation, suppressing IGF signaling	Lui et al., 2016
	NSD1	H3K36me2		Human	Mutations cause Sotos syndrome with overgrowth phenotypes	Qiao et al., 2011
	NSD2	H3K36me3	*Opn, Col1a1*	Human	Mutations cause Wolf-Hirschhorn syndrome with overgrowth phenotypes	Lee et al., 2014
	CARM1/PRMT4	Arginine methylation of *Sox9*	*Sox9*	Murine	Promoting chondrocyte proliferation and differentiation	Ito et al., 2009
	PRMT5	?	*Col2A1, Col10A1, Prg4*	Murine	Promoting chondrocyte hypertrophy	Ramachandran et al., 2019

**TABLE 2:** Histone-modifying enzymes serve as erasers to regulate chondrocyte differentiation.

	Enzyme	Histone/target	Genes regulated	Species	Effects on chondrocyte function	References
HDAC	HDAC1	?	*Comp*	Murine	Repressing transcription of *Comp*	Liu et al., 2007
		H3K9	*UDP-glucose dehydro -qenase (Uqdh)*	Rat	Inhibiting transcription of *Ugdh*	Wen et al., 2020
		?	*β-catenin*	Human	Repressing transcription of β-catenin, downregulating the canonical Wnt signaling	Huang et al., 2014
		H3K9, H3K14	*Sox9*	Murine	Repressing *Sox9*	Tie et al., 2018
	HDAC2	H3	*COMP, ACAN, COL2A1*	Human	Repressing transcription of *COMP, ACAN, COL2A1*	Mao et al., 2017
	HDAC2	H3K9	*Tgfβr1*	Rat	Inhibiting cartilage matrix synthesis	Li et al., 2020
	HDAC2/8	H3	*COL2A1*	Human	Repressing *COL2A1*	Chen W. et al., 2016
	HDAC3	H3K9/K14	*Phlpp1*	Murine	Repressing transcription of *Phlpp1*, upregulating Akt signaling	Bradley et al., 2013
		H3	*COL2A1, ACAN, COMP, SOX9*	Human	Repressing transcription of *COMP*, *ACAN*, COL2A1, and SOX9	Meng et al., 2018
	HDAC4	H3	*Runx2*	Murine	Repressing Runx2, and itsbinding to target genes; mutations cause brachydactyly type E	Vega et al., 2004; Williams et al., 2010
	HDAC4, HDAC5	?	*Mef2, Runx2*	Murine	Regulating chondrocyte hypertrophy	Nishimori et al., 2021
	HDAC7	?	*MMP13*	Human	Promoting expression of *MMP13*	Higashiyama et al., 2010
	HDAC8	SMC3 deacetylase	*Cohesin*	Human	Loss-of-function mutation causes Cornelia de Lange syndrome with upper limb anomalies	Deardorff et al., 2012
	HDAC9	?	*Nkx3.2*	Murine Human	Regulating chondrocyte survival and hypertrophic maturation	Choi et al., 2016
	HDAC?	H3K9/14	*Leptin*	Human	Repressing transcription of leptin, inhibiting MMP13 activity	Iliopoulos et al., 2007
	HDAC? (except 1,6,11)	H4	*Wnt5a*	Rabbit	Repressing transcription of Wnt5a, promoting expression of type II collagen	Huh et al., 2007
KDM	LSD1 (KDM1A)	H3K4me2	*Sox9*	Murine	Repressing *Sox9*	Zhang et al., 2016b
		H3K4me2	*Nfat1*	Murine	Repressing *Nfat1*, inhibiting *Acan*, *Col2A1, Mmp13*	Zhang et al., 2016a
		H3K9me1/2	*mPGES-1*	Human	Promoting expression of mPGES-1, contributing to the biosynthesis of PGE2.	El Mansouri et al., 2014
	KDM4B (JMJD2B)	H3K9me3	*SOX9*	Human	Promoting expression of SOX9	Lee et al., 2016
	UTX (KDM6A)	H3K27me3	*SOX9*	Human	Promoting expression of *SOX9, COL2A1, ACAN*	Wang et al., 2018a
	JMJD3 (KDM6B)	H3K27me3	*Runx2, Ihh*	Murine	Promoting expression of *Runx2, Ihh*	Zhang F. et al., 2015
			*Col2A1, Acan*	Murine	Promoting expression of *Col2A1, Acan*	Dai et al., 2017
		?	*Sox9,Col2A1, Acan*	Murine	Promoting expression of *Sox9,Col2A1, Acan*	Huang et al., 2020
	PHF2	H3K9me2	*Col2A1, Acan*	Murine	Phf2 knockdown inhibits Sox9-induced chondrocyte differentiation	Hata et al., 2013

**TABLE 3:** Proteins or domains serves as readers to regulate chondrocyte function.

	Protein or domain	Name	Histone	Genes regulated	Species	Effects on chondrocyte function	References
Acetylation readers	Bromodomain	BRD4		*HMGB1*	Human	Enhancing *HMGB1* gene transcription	Jiang et al., 2017
			H3K27ac	*Col1A1*	Murine	Upregulating *Col1A1* expression	Ding et al., 2015
		BRD3, BRD4	H4K5/8/12 ac	*MMP1, MMP3, ADAMTS4*	Human	Promoting expression of matrix-degrading enzyme genes	Dai et al., 2019
Methylation readers	Set/Ash HMT complex	WDR5	H3K4me3	*Twist-1*	Murine	Promoting chondrocyte proliferation and differentiation	Gori et al., 2009
	PHD fingers	PHD fingers	H3K4me	*Trim24*	Murine	Regulating TRIM24-RIP3 pathway and OA pathogenesis	Jeon et al., 2020
	Ankyrin repeats	Ankyrin repeats		*Col10A1, Mmp13*	Murine	Suppressing chondrocyte proliferation and differentiation	Nakatomi et al., 2019

## Writers Regulating Chondrocyte Fate

### Histone Acetyltransferases (HATs)

The enzymes that catalyze histone acetylation are referred to as HATs. Early studies identify the involvement of histone acetylation in transcriptional regulation of gene expression (Sterner and Berger, 2000). According to the ‘charge hypothesis’, positively charged unmodified histones bind negatively charged DNA via electrostatic interactions to form nucleosomes. In general, within this tightly packed structure, genes are in a repressed state (Michael, 1997). Acetylation by HATs negatively charges the lysine residues in the N-terminal tail of histones extending from the nucleosome, allowing the chromatin structure to relax, which increases the accessibility of RNA polymerase II (RNAP II), enables the binding of transcription factors, and subsequently facilitates gene transcription (Roth et al., 2001; Park and Kim, 2020). It is well established that HATs are actively involved in the regulation of chondrocyte differentiation through enhancing the expression of chondrogenic marker genes, such as *SOX9*, *COL2A1*, and *COMP* (Table 1).

Early studies show that E1A (Adenovirus early region 1A)-associated protein p300 (EP 300 or p300) and cAMP-response element-binding protein (CREB)-binding protein (CBP) act as HATs to regulate chromatin activity through the modulation of histone acetylation (Bannister and Kouzarides, 1996; Ogryzko et al., 1996; Chan and La-Thangue, 2001). In addition, p300/CBP function as important transcriptional co-activators and are involved in multiple, signal-dependent transcription events to regulate cell proliferation, differentiation and apoptosis. Mechanistically, p300/CBP act as protein bridges that connect different sequence-specific transcription factors to the transcription apparatus, or serve as a protein scaffold to build a multicomponent transcriptional regulatory complex. Early studies indicate that CBP is required for CREB-mediated gene expression (Korzus et al., 1998). Heterozygous mutations of p300 or CBP cause Rubinstein-Taybi syndrome (RTS) (Petrij et al., 1995; Bartholdi et al., 2007; Negri et al., 2015), a rare autosomal dominant genetic disease, manifested by short stature, psychomotor and mental retardation, that was firstly reported in 1963 (Rubinstein and Taybi, 1963). This suggests that p300/CBP may be involved in the regulation of longitudinal development of long bones controlled by growth plate chondrocytes. In the chondrosarcoma SW1353 cells, SOX9 is found to associate with p300/CBP through its carboxyl terminus activation domain in a cell type-specific manner (Tsuda et al., 2003), hyperacetylating histones H3 at lysine 9 (H3K9) and H4 at lysine 8 (H4K8) around the *COL2A1* enhancer region (Furumatsu et al., 2005). The SOX9/p300/CBP activator complex binds to the positive regulatory element (PRE) in the proximal region of the promoter of target genes, which results in loosening of the chromatin structure and activation of *COL2A1* and *COMP* gene expression (Liu et al., 2007). Mutation of COMP causes pseudoachondroplasia and multiple epiphyseal dysplasia (Briggs et al., 1995). As an ECM protein, COMP serves as a marker of cartilage turnover (Petersen et al., 2010). These studies indicate that p300/CBP serve as important HATs to regulate chondrocyte fate and functions.

General control non-derepressible 5 (GCN5) or KAT2A is another HAT that is required for murine and zebrafish craniofacial development. Genetic disruption of *Gcn5* in zebrafish embryos inhibits the expression of cartilage specific marker genes including *Sox9* and *Col2A1*, accompanied by a reduction in the H3K9 acetylation (H3K9ac) level (Sen et al., 2018). However, a recent study show that deletion of *Gcn5* in mice decreases craniofacial chondrocyte size and maturation compared with the control mice. GCN5 acts through activation of mTORC1 or indirect mechanisms rather than as an epigenetic regulator of H3K9ac. GCN5 is not required for H3K9ac at *Acan* enhancers specific for pre-hypertrophic chondrocytes (Pezoa et al., 2020). These results suggest the complexity of GCN5 in regulation of skeletal development and distinct mode of actions in different species.

Most HATs exist in multi-protein complexes, and it is the associated proteins that allow the enzymes to identify the correct site to be modified (Lee and Workman, 2007). So, to explain how HATs carry out specific functions in the cells even if they do not have unique substrates, it is essential to define the context they exist.

### Histone Methyltransferases (HMTs)

Histone methylation occurs at lysine, arginine and histidine residues. The most common methylation occurs at histones H3 and H4, particularly at H3K4 and H3K9 (Greer and Shi, 2012). Histone methylation involves addition of methyl groups to the amino acid lysine in the histone tail. More than one lysine can be methylated on the same histone. The enzymes that catalyze histone methylation are referred to as HMTs (Zhang and Reinberg, 2001; Hyun et al., 2017; Husmann and Gozani, 2019), a panel of which are shown to regulate chondrocyte functions (Table 1). Lysine methylation is involved in both transcriptional activation and repression, depending on the site of methylation. Modifications associated with transcription activation include histone 3 lysine 4 methylation (H3K4me), histone 3 lysine 36 methylation (H3K36me), histone 3 lysine 79 methylation (H3K79me), whereas modifications associated with gene silencing or repression include histone 4 lysine 20 methylation (H4K20me), histone 3 lysine 27 methylation (H3K27me), and histone 3 lysine 9 methylation (H3K9me) (Martin and Zhang, 2005; Edmunds et al., 2008; Zhang T. et al., 2015). This flexibility can be explained by the fact that methylation does not alter histone charge but directly affect histone-DNA interactions. Residues of histones can also be mono-, di-, or tri-methylated, and each degree of modification is associated with different biological responses, thus providing further functional diversity for each methylation site (Lanouette et al., 2014). An alternative mode of cooperation may also exist among HMTs. For instance, in mammals, heterochromatic regions are highly trimethylated on H3K9, whereas silent euchromatin regions are enriched for mono- and di-methylated H3K9, negatively regulating gene expression (Wang et al., 2003; Martin and Zhang, 2005). H3K9me has been shown to be involved in the development of OA in the temporomandibular joint, where H3K9me level is decreased in the degenerated condylar articular cartilage of aged mice (Ukita et al., 2020). Interestingly, the levels of H3K9me3 and SUV39H1 are elevated in chondrocytes from the facet joint cartilage of idiopathic scoliosis patients compared with the control group, which might supress the miR-15a/Bcl2 axis to facilitate chondrocyte proliferation and abnormal spinal growth (Li et al., 2019). This indicates that SUV39H1 may participate in the pathogenesis of facet joint cartilage during development and progression of idiopathic scoliosis.

Histone lysine *N*-methyltransferase 1 (Ehmt1), also known as G9a-like protein (GLP), and Ehmt2 (also known as G9a) are mainly responsible for mono- and di-methylation of H3K9 in euchromatin (Tachibana et al., 2005). The suppressor of variegation 3-9 (drosophila) homolog 1 (SUV39H1) and Su(var)3-9, Enhancer-of-zeste and Trithorax (SET) domain bifurcated histone lysine methyltransferase 1 (SETDB1), also known as ERG-associated protein with SET domain (ESET) are identified as the essential H3K9 trimethylases. However, trimethylation at H3K9 (H3K9me3) can neither be achieved by a single step from a non-methylated state, nor can it be accomplished independently by a single trimethylase. SUV39H is suggested to catalyze H3K9me based on the mono- and di-methylation regulated by G9a. On the other hand, SETDB1-mediated conversion of H3K9me to H3K9me3 requires the facilitation by the human homolog of mAM (mouse ATFa-associated Modulator) (Peters et al., 2003; Wang et al., 2003). hAM, which is also known as activating transcription factor 7-interacting protein (ATF7IP), or methyl-CpG binding domain (MBD) 1-containing chromatin-associated factor (MCAF). ATF7IP is shown to regulate the spatial or temporal recruitment of SETDB1 to target sites on the chromatin (Basavapathruni et al., 2016), and plays critical role in heterochromatin formation by regulating SETDB1 stabilization in the nucleus (Timms et al., 2016). ATF7IP mediates SETDB1 retention inside the nucleus, presumably by inhibiting its nuclear export by binding to the N-terminal region of SETDB1, which harbors the nuclear export signal motifs, and by promoting its nuclear import (Tsusaka et al., 2019). This suggests that ATF7IP regulates SETDB1 activity accompanied by its nuclear translocation.

The four main H3K9 HMTs, Ehmt1, Ehmt2, SETDB1, and SUV39H1, are suggested to coexist in the same complex and regulate the target genes in a coordinated manner (Fritsch et al., 2010). SETDB1/ESET is shown to be essential to the hypertrophic differentiation of growth plate chondrocytes and formation of the epiphyseal plates (Yang et al., 2013). ESET is also involved in the regulation of RUNX2 (Lawson et al., 2013a), the key transcription factor controlling osteoblast differentiation, inducing chondrocyte hypertrophy and upregulating matrix-degrading enzymes such as matrix metalloproteinases (MMPs) and a disintegrin and metalloproteinase with thrombospondin motifs (ADAMTs) (Zhao et al., 2016). A transient upregulation of ESET is observed in prehypertrophic chondrocytes in newborn mice. Conditional deletion of *Eset* (exons 15–16) in mesenchymal cells using Prx1 promoter driven Cre recombinase (Prx1-Cre) accelerates chondrocyte hypertrophy, causes disorganization of growth plate chondrocytes, and impairs long bone growth. Mechanistically, ESET interacts with RUNX2-histone deacetylases 4 (HDAC4) and suppresses RUNX2-mediated osteocalcin gene transactivation. ESET is also required for normal production of IHH in prehypertrophic and early hypertrophic chondrocytes (Yang et al., 2013). Conditional deletion of Eset in mesenchymal cells results in hypertrophy, apoptosis, and terminal differentiation of articular chondrocytes. This is accompanied by an upregulation of MMP13 and alkaline phosphatase (ALP) activity, the markers of hypertrophic chondrocytes (Lawson et al., 2013b). Interestingly, exon 4 deletion of *Eset* using the same Cre-loxp strategy results in decreased thickness of articular cartilage in E17.5 mouse embryos, whereas deletion of exons 15–16 does not show the same phenotype (Yang et al., 2018). siRNA mediated SETDB1 knockdown leads to downregulation of chondrogenic gene expression, including *SOX9*, *ACAN*, *COL2A1*, and *MMP13*, in chondrocytes from Meckel’s cartilage, from which the mandibles are derived, suggesting that other mechanisms besides the HMT activity may also involve in its regulation, which awaits further elucidation (Yahiro et al., 2017). GLP contributes to the inhibition of *Runx2* through H3K9me2 at the *Runx2* promoter (Balemans et al., 2014).

SET domain containing-1A (SETD1A) expression is elevated in OA cartilage. H3K4 di- or tri-methylation at the *iNOS* and *COX-2* promoters is conducted by SETD1A, contributing to IL-1-induced *iNOS* and *COX-2* expression (El Mansouri et al., 2011). COX-2 is the key enzyme for the biosynthesis of prostaglandin E_2_ (PGE_2_). COX-2 expression and activity are increased in articular cartilage from patients with OA, which is thought to play a primary role in pain and inflammation associated with the disease (Martel-Pelletier et al., 2003). In addition, nitric oxide (NO) participates in both rheumatoid arthritis (RA) and OA by inducing chondrocyte apoptosis and MMPs production, and by suppressing the synthesis of collagen and proteoglycans. NO also enhances the production of inflammatory cytokines and PGE_2_ (Sasaki et al., 1998). These findings suggest a potential link between SETD1A and NO mediated regulation in chondrocytes that deserves to be further defined.

The PRDM (PRDI-BF1 and RIZ homology domain containing) proteins are characterized by consisting of an N-terminal PR (PRDI-BF1 and RIZ1 homology) domain, which shares high homology with the catalytic SET domain that defines a group of HMTs (Xiao et al., 2003). In the human genome, 17 *PRDMs* (*PRDM 1–17*) are identified to encode proteins with a PR/SET. Except for PRDM11, all other PRDMs have a variable number of Zn-finger domains (Fumasoni et al., 2007). Loss of *Prdm3* and *Prdm16* reduces methylation of H3K9 and H3K4 in zebrafish, leading to craniofacial defects, including hypoplasia of the craniofacial cartilage elements, undefined posterior ceratobranchials, and decreased mineralization of the parasphenoid. Deletion of *Prdm3* inhibits *Sox9* and *Col2A1* expression in the pharyngeal skeleton, suggesting an overall loss of chondrocyte functionality. Meanwhile, in mice, loss of *Prdm16* significantly decreases H3K9 methylation but not H3K4 methylation in the palatal shelves, and causes craniofacial defects, including anterior mandibular hypoplasia, clefting in the secondary palate and severe middle ear defects (Shull et al., 2020). These data indicates that PRDMs participate in the regulation of bone and cartilage development.

GWAS and functional studies identify the disruptor of telomeric silencing 1-like (*DOT1L*) gene to be involved in cartilage thickness and hip OA. Reducing DOT1L expression in chondrogenic cell lines suppresses *COL2A1*, *COL10A1*, and *ACAN* gene expression but increases expression of osteogenic genes, such as *COL1A1* (Castaño Betancourt et al., 2012). H3K79 methylation, as well as DOT1L, is reduced in damaged areas of OA joints (Monteagudo et al., 2017). DOT1L is found to prevent Wnt hyperactivation by suppressing the activity of SIRT1 and methylating H3K79 in the lymphoid enhancer-binding factor-1 (*Lef1*) and T cell factor-1 (*Tcf1*) promoters, which are Wnt target genes, thereby decreasing *Col10A1* and *Mmp13* expression, as well as maintaining cartilage homeostasis (Monteagudo et al., 2017). Activation of Wnt signaling is suggested to stimulate chondrocyte hypertrophy and inhibit chondrogenesis, which has been considered as a risk factor for OA (Usami et al., 2016). These findings are consistent with what is observed in mice with deletion of *Dot1l* in chondrocytes, characterized by reduction in proteoglycan component of the ECM (e.g., chondroitin sulfate), decreased chondrocyte proliferation and accelerated OA progression (Cornelis et al., 2019; Jo et al., 2020). Mice lacking *Dot1l* in mesenchymal progenitors exhibit skeletal dysplasia characterized by limb shortening, abnormal bone morphologies and forelimb dislocations (Sutter et al., 2021). These results suggest that DOT1L plays crucial role in regulating chondrocyte proliferation, differentiation, ECM production and endochondral bone formation.

Absent, small, or homeotic 1 (Ash1)-like (Ash1l), a member of the trithorax group (TrxG) proteins, contributes to the formation of H3K4me3 and H3K36me3 in the promoter-proximal coding region of target genes (Miyazaki et al., 2013; Xia et al., 2017). It is found that *Ash1l* silencing in mice leads to severe cartilage and bone destruction compared with the wild type controls (Xia et al., 2013). Moreover, Ash1l is suggested to promote chondrogenic differentiation of MSCs through upregulation of H3K4me3 at the *Runx2*, *Homeobox a10* (*Hoxa10*), and *Sox9* gene promoters (Yin et al., 2019). Along with other genes crucial to cartilage development, including *RUNX2* and *SOX9*, *HOXa10* is also associated with cartilage pathology and its expression tends to be decreased in OA chondrocytes (Pelttari et al., 2015).

Another H3K4 methylase, lysine methyltransferase 2D (KMT2D), also regulates SOX9 expression, but through an indirect way. KMT2D elevates H3K4me3 at the promoter of short stature homeobox 2 (*Shox2*) gene, promotes *Shox2* transcription and subsequently inhibits *Sox9* expression, leading to impaired chondrocyte differentiation through inactivation of *Col2A1* and *Col10A1* genes (Fahrner et al., 2019). As a well-known regulator of cytokine expression during the immune response, nuclear factor of activated T cells 1 (NFAT1) exhibits an age-dependent expression in mouse articular cartilage. A simultaneous increase in NFAT1 and H3K4me2 expression is observed in embryonic articular chondrocytes. However, NFAT1 expression is decreased in 6-month-old articular chondrocytes with corresponding increased H3K9me2 activity and transcriptional repression (Zhang et al., 2016a). This relationship suggests the critical role of histone methylation in age-related NFAT1 expression and thereby may play a role in epigenetic modifications in the pathogenesis of OA.

The enhancer of zeste homolog 1/2 (EZH1/2), embryonic ectoderm development (EED) and suppressor of zeste 12 homolog (SUZ12) are core components of the polycomb repressive complex 2 (PRC2), which catalyzes H3K27me3 and silences chromatin (Simon and Kingston, 2009). Deficiency of these components may impair the HMT activity of PRC2. Loss of *Ezh2* in mouse MSCs results in a neonatal phenotype of shorter limbs and vertebrae due to premature maturation of the epiphysis (Schwarz et al., 2014). Mice with double knockout of *Ezh1* and *Ezh2* in chondrocytes show severely impaired growth plate chondrogenesis indicated by deceased chondrocyte proliferation and hypertrophy. The decreased chondrocyte proliferation is partially associated with derepression of cyclin-dependent kinase inhibitors Ink4a/b, while the impaired chondrocyte hypertrophy is due to the suppression of IGF signaling by upregulation of IGF-binding proteins (Lui et al., 2016). In addition, *Ezh2* deletion in chondrocytes causes reduced H3K27me3 level, and leads to enhanced expression of osteogenic factors including BMP2, BMP2 responsive transcription factor Sp7 (Osterix), and osteoblast specific ECM proteins such as integrin binding bone sialoprotein (IBSP) and osteocalcin. Nevertheless, joint development proceeds normally without the appearance of excessive hypertrophy or premature OA phenotype. This phenotype indicates that EZH2 activity is dispensable for chondrocyte maturation and endochondral ossification (Camilleri et al., 2018). In addition, deletion of *Eed* in chondrocytes causes severe kyphosis and growth defect with decreased chondrocyte proliferation, accelerated hypertrophic differentiation and cell death. The phenotypes are associated with reduction of HIF-1α expression, overactivation of Wnt and TGF-β signaling (Mirzamohammadi et al., 2016). Mutations in the *EZH2* gene in human are associated with Weaver syndrome, a rare genetic disease characterized by advanced osseous maturation, skeletal and neurological abnormalities (Gibson et al., 2012). It is similar to Sotos syndrome that is caused by mutations of the nuclear receptor-binding SET domain 1 (NSD1) gene (Douglas et al., 2003). Both EZH2 and NSD1 catalyze methylations of H3K36 and H3K27, suggesting that mutations in writers of these two chromatin marks might cause overgrowth conditions, resembling Sotos or Weaver syndromes. Indeed, heterozygous mutations of SETD2 gene are identified in patients with Sotos-like syndrome (Luscan et al., 2014). These findings suggest that PRC2 plays critical roles in the regulation of chondrocyte function and endochondral bone formation.

In addition to its role in endochondral bone growth, EZH2 is shown to play important roles in the pathogenesis of OA. EZH2 is significantly upregulated in articular chondrocytes of OA patients and OA mouse model when compared with that of the normal controls (Chen L. et al., 2016; Allas et al., 2020). Overexpression of EZH2 increases *Ihh*, *Mmp13*, *Adamts-5*, and *Col10A1* expression, while inhibition of EZH2 suppresses these genes expression in chondrocytes. Induction of *EZH2* activates β-catenin signaling by increasing H3K27me3 on the promoter of secreted frizzled-related protein 1 (*Sfrp1*) gene, a modulator of Wnt. Conditional deletion of *Ezh2* in chondrocytes deteriorates OA pathological changes in the medial meniscectomy induced mouse OA model, as indicated by inhibition of chondrocyte hypertrophy through activating tumor necrosis factor ligand superfamily member 13B (TNFSF13B) (Du et al., 2020). These studies indicate that EZH2 may serve as an effective target for OA therapy.

As H3K36-specific HMTs, the NSD family proteins (NSD1, NSD2, and NSD3) are the main contributors of H3K36me2 (Li et al., 2009; Kuo et al., 2011; Qiao et al., 2011; Huang and Zhu, 2018). Haploinsufficiency of NSD1 gene causes Sotos syndrome (Kurotaki et al., 2002; Douglas et al., 2003; Melchior et al., 2005). Children with Sotos syndrome exhibit overgrowth phenotypes, characterized by being taller and heavier, and giving larger body size and relatively large skulls (macrocephaly) than those normal children at the same age. This suggests that NSD1 controls the activity of genes involved in skeletal growth and development. Tatton Brown-Rahman syndrome (TBRS), another childhood overgrowth disorder that is defined by germline mutations in DNA (cytosine-5)-methyltransferase 3A (*DNMT3A*), shares similar clinical features with Sotos syndrome. Both H3K36me2 and H3K36me3 recognize the PWWP domain of DNMT3A protein, with a higher binding affinity toward H3K36me2. These observations reveal a *trans*-chromatin regulatory pathway that connects aberrant intergenic CpG methylation to human neoplastic and developmental overgrowth (Weinberg et al., 2019). Interestingly, mice carrying a D329A point mutation in the *Dnmt3a* PWWP domain exhibit dominant postnatal growth retardation. Mechanistically, this mutation targets H3K27me3 and bivalent chromatin-marked domains for DNA hypermethylation, which leads to loss of repression of transcription factor genes controlling development and altered histone PTM landscape. The growth deficit phenotypes of the heterozygous and homozygous mutants are not associated with reduced serum levels of GH or IGF-1, the major modulators of postnatal growth (Sendžikaitė et al., 2019). These findings indicate that mutations in the DNMT3A PWWP domain may cause growth retardation by altering histone PTMs, while detailed molecular mechanisms in different cell lineages deserve to be further defined. It is noteworthy that through recognition of histone H3K36me3 mark, DNMT3A (Xu et al., 2020) and DNMT3B (Baubec et al., 2015; Neri et al., 2017) play critical roles in mediating *de novo* DNA 5mC in different genomic regions. This indicates that different histone modifications may crosstalk to the establishment of *de novo* DNA 5mC patterns.

Mutation of H3K36 trimethyltransferase *WHSC1* gene (Wolf–Hirschhorn Syndrome candidate 1, also known as *NSD2*) is the major cause of Wolf–Hirschhorn syndrome (WHS), characterized by developmental defects including growth delay, mental retardation, short stature, radioulnar synostosis and mesomelic limb shortness, and craniofacial abnormalities (Bergemann et al., 2005; Mazzeu et al., 2007). WHSC1 is crucial in regulating the transcriptional activation of bone-related genes, including *OPN* and *COL1A1*, through its association with RUNX2 and p300 (Lee et al., 2014). These clinical and experimental data suggest that NSD2 is an important regulator controlling skeletal longitudinal growth and bone formation.

H3K36M (replacing lysine-36 with methionine-36) and H3K36I (replacing lysine-36 with isoleucine-36) mutations can drive tumorigenesis of chondroblastoma by inhibiting H3K36 methyltransferases (e.g., MMSET and SETD2) and reprogramming the H3K36 methylation landscape, and are thus considered as oncohistones. H3K36M substantially inhibits chondrocyte differentiation indicated by decreased mRNA levels of chondrocyte markers *COL2A1*, *COL9A1*, *COL11A1*, and *ACAN* (Fang et al., 2016; Lu et al., 2016). Transgenic mice with engineered H3K36M mutation not only show global reduction in H3K36me2 accompanied by a collapse of normal H3K27me3 distribution, but also have defects in chondrocyte differentiation (Abe et al., 2020). This highlights an importance of the balanced regulation between H3K36me2 and H3K27me3 during chondrocyte differentiation.

Histone arginine methylations are also involved in the regulation of chondrogenesis. Three main forms of methylated arginine have been identified in eukaryotes: ω-*N*^*G*^, monomethylarginines (MMA); ω-*N*^*G*^, *N*^*G*^-asymmetric dimethylarginines (aDMA); and ω-*N*^*G*^, *N*^*G*^-symmetric dimethylarginines (sDMA) (Bedford and Richard, 2005). Arginine methylations are catalyzed by protein arginine methyltransferases (PRMTs). The PRMTs share a core region composed of a conserved Ado-Met binding domain and a more divergent C-terminal domain. Currently, eight mammalian PRMTs have been identified. PRMTs are classified as either type I or type II enzymes. Both types catalyze the formation of MMA as an intermediate, and type I PRMTs (PRMT1, PRMT3, PRMT4, and PRMT6) lead to the production of aDMA, whereas type II PRMTs (PRMT5 and PRMT7) catalyze the formation of sDMA (Bedford and Richard, 2005). Among the PRMTs, PRMT4 and PRMT5 are shown to be involved in the regulation of chondrogenesis and long bone development. Coactivator-associated arginine methyltransferase 1 (CARM1)/PRMT4 specifically methylates *Sox9* at its HMG domain and disrupts the interaction of SOX9 with β-catenin, which subsequently regulates Cyclin D1 expression and cell cycle progression of chondrocytes (Ito et al., 2009). Mice lacking *Prmt5* in the limb bud mesenchyme (Prx1-Cre mediated deletion) have severely truncated bones with wispy digits lacking joints, which is caused by widespread cell death of mesodermal progenitor cells (Norrie et al., 2016). Mice lacking *Prmt5* in chondrocytes exhibit a striking blockage in hypertrophic chondrocyte differentiation and abnormal expression of chondrogenic marker genes (Ramachandran et al., 2019). These observations suggest that histone arginine methylations play an important role in chondrocyte proliferation, differentiation and homeostasis during chondrogenesis, but the potential involvement of PRMTs in various chondropathies remain to be explored.

## Erasers Regulating Chondrocyte Differentiation

### Histone Deacetylases (HDACs)

The enzymes that catalyze the removal of an acetyl group from a histone are called HDACs. Eighteen HDACs have been identified in humans. Based on the function and DNA sequence homology to yeast original enzymes and domain organization, the 18 human HDACs are grouped into four classes: classes I, II, III (sirtuins or SIRTs) and IV. Class I (HDAC 1, 2, 3, and 8), II (HDAC 4, 5, 6, 7, 9, and 10), and IV (HDAC 11) enzymes are sub-grouped as “classical” HDACs, which possess a zinc dependent active site and can be inhibited by trichostatin A (TSA), whereas Class III enzymes (SIRT 1, 2, 3, 4, 5, 6, and 7) are nicotinamide adenine (NAD^+^)-dependent and mostly not affected by TSA (Dokmanovic et al., 2007; Bradner et al., 2010). HDACs have been shown to exert both histone modification and none-histone effects. A large body of studies on HDACs indicate that HDACs play important roles in regulating chondrocyte differentiation and functions (Table 2).

Germline deletion of *Hdac1* in mice causes embryonic lethality before E10.5 due to severe proliferation defects and developmental retardation. The phenotypes are associated with significantly reduced overall HDAC activity, hyperacetylation of a subset of histones H3 and H4, and alterations in other histone modifications. Deletion of *Hdac1* induces the expression of *Hdac2* and *Hdac3*, but cannot compensate for the entire function of *Hdac1* (Lagger et al., 2002). Using HDAC1-deficient embryonic stem cells (ESCs) as a model, it is revealed that there exists a regulatory cross talk between HDAC1 and HDAC2, and HDAC1 may serve as an important transcriptional coactivator (Zupkovitz et al., 2006). Interestingly, deletion of *Hdac1*, but not *Hdac2*, causes a significant reduction in the HDAC activity of Sin3A, NuRD, and CoREST corepressor complexes (Dovey et al., 2010). This suggests that HDAC1 exerts unique functions during embryogenesis as well as ESC differentiation. In an *in vitro* chondrogenesis model, HDAC1 is shown to be required for the repressive effects of leukemia/lymphoma related factor (LRF) on BMP-2-induced chondrogenesis in micromass cultures of C3H10T1/2 cells. LRF recruits HDAC1 to the negative regulatory element (NRE) of the *Comp* gene promoter and downregulates *Comp* transcription (Liu et al., 2004). In addition, HDAC1 directly binds to the promoter of β-catenin gene (*Ctnnb1*) through its deacetylase domain to suppress *Ctnnb1* expression (Huang et al., 2014). Canonical Wnt signaling is mediated by β-catenin, a key modulator controlling gene transcription in chondrocytes (Usami et al., 2016). In the zebrafish HDAC1(b382) mutants, craniofacial cartilage development is defective due to apoptosis of chondroprogenitors in the posterior branchial arch and impaired chondrocyte differentiation (Ignatius et al., 2013). This provides direct evidence of HDAC1 in regulating chondrocyte survival and differentiation in zebrafish. Maternal exposure to dexamethasone induces impaired growth plate development with decreased proteoglycan content. The phenotype is associated with H3K9 acetylation of uridine diphosphate (UDP)-glucose dehydrogenase (*Ugdh*) gene, which encodes a key enzyme controlling proteoglycan synthesis in chondrocytes. Mechanistically, dexamethasone induces the binding of glucocorticoid receptor (GR) to the *Ugdh* gene promoter, which recruits HDAC1 and Specific protein 3 (Sp3), induces H3K9 deacetylation, and subsequently inhibits Ugdh gene expression and proteoglycan synthesis (Wen et al., 2020). These data suggest that HDAC1 is involved in regulation of chondrocyte differentiation and ECM production. An *in vitro* assay indicates that nicotine increases the binding of NFATc2 and HDAC1 on the *Sox9* promoter, while decreasing the H3K9ac and H3K14ac levels on the *Sox9* promoter. In a rat osteochondral defect repair model, nicotine impairs articular cartilage repair following transplantation of bone marrow MSCs when compared with the control group (Tie et al., 2018). These results indicate that the negative impact of nicotine on bone marrow MSCs mediated articular cartilage repair might be associated with alterations of HDAC1 regulatory effect, but detailed mechanisms remain yet to be elucidated.

Both HDAC1 and HDAC2 are upregulated in articular chondrocytes of patients with OA (Hong et al., 2009; Mao et al., 2017). Two carboxy-terminal domains (CTDs) of HDAC1 and HDAC2 are identified to interact with the Snail transcription factor to suppress *COL2A1* gene expression in human chondrocytes, but independent of the enzymatic activities of both HDACs (Hong et al., 2009). The upregulation of HDAC2 is accompanied by decreased microRNA-92a-3p (miR-92a-3p) level in human OA chondrocytes. miR-92a-3p is found to directly target the 3′-UTR of HDAC2 mRNA, and serves as a suppressor of HDAC2. miR-92a-3p enhances acetylation of H3 on the promoters of *ACAN*, *COMP* and *COL2A1* (Mao et al., 2017). Interestingly, prenatal caffeine exposure (PCE) induces OA susceptibility in male adult offspring rats. The phenotype is associated with elevation of serum corticosterone levels, which reduces the H3K9ac level on *Tgfβr1* gene promoter region through acting on GR and recruiting HDAC2 into the nucleus of chondrocytes (Li et al., 2020). These results suggest that HDAC2 coordinates with GR to regulate histone acetylation levels on key regulators (i.e., TGFβR1) controlling chondrocyte fate.

Mice with conditional deletion of *Hdac3* in osterix-expressing progenitors have decreased bone density and increased adipogenesis in the bone marrow, which is accompanied by delayed or arrested terminal differentiation of growth plate chondrocytes and impaired endochondral bone formation (Razidlo et al., 2010). *Hdac3*-deficient chondrocytes are smaller in size and enter hypertrophy earlier than normal chondrocytes, accompanied by reduced production and secretion of sulfated glycosaminoglycan, aggrecan, osteopontin, as well as ECM phosphoglycoprotein (Bradley et al., 2013). HDAC3 localizes to the promoter of PH domain leucine-rich repeat protein phosphatase 1 (*Phlpp1*), decreases H3 K9/K14 acetylation and represses *Phlpp1* expression in a TGFβ-regulated manner (Bradley et al., 2013). Repressed *Phlpp1* expression enhances the phosphorylation of AKT2, PKC, and p70s6 kinase, and increases chondrocyte proliferation and hypertrophy (Bradley et al., 2013, 2015a). Further, in a mouse OA model, *Phlpp1* deficiency protects against OA progression by regulating *Phlpp1* promoter CpG methylation in cartilage (Bradley et al., 2016).

The expression levels of *SOX9*, *COL2A1*, *ACAN*, and *COMP* are shown to be downregulated by HDAC3, mediated by enhancement of H3 deacetylation at the promoter of these genes. In an analyses of OA articular cartilage samples, the relative expression level of HDAC3 in degraded articular cartilages is higher than that of the non-degraded ones. This is accompanied by decreased miR-193b-3p level (Meng et al., 2018). These data provide new insights into the regulation of chondrogenesis and OA pathology by HDAC3.

HDAC4 is abundantly expressed in pre-hypertrophic chondrocytes and plays a central role in regulating chondrocyte hypertrophy (Vega et al., 2004; Chen et al., 2020). Mice lacking *Hdac4* exhibits early onset chondrocyte hypertrophy and premature endochondral ossification. HDAC4 physically interacts with *Runx2* and consequently inhibits *Runx2* DNA binding and transcriptional activity (Vega et al., 2004). Mice lacking *Hdac4* in chondrocytes phenocopy mice with germline mutation of *Hdac4* (Nishimori et al., 2019a). In addition, HDAC4 is found to interact with the myocyte enhancer factor MEF2A in the nucleus, resulting in the repression of MEF2A transcriptional activation (Miska et al., 1999). HDAC4 coordinates with MEF2C to regulate chondrocyte hypertrophy and endochondral bone formation (Arnold et al., 2007). It is well known that PTHRP functions as a paracrine factor to regulate chondrocyte proliferation and suppress its hypertrophy (Karaplis et al., 1994; Kronenberg, 2003). PTHRP action results in dephosphorylation of HDAC4, and drives its nuclear translocation in chondrocytes (Kozhemyakina et al., 2009; Nishimori et al., 2019a). This is associated with reduction of HDAC4 phosphorylation at the binding sites of 14-3-3 protein, which subsequently represses chondrocyte hypertrophy and bone formation by blocking MEF2/RUNX2 signaling cascade. HDAC5 is also involved in this regulatory process, but in a lesser degree (Nishimori et al., 2019a). Interestingly, HDAC4 and HDAC5 serve as key PTH1R-regulated salt inducible kinase 1 (SIK1) substrates to repress chondrocyte hypertrophy (Nishimori et al., 2019b, 2021). SIK1, a subclass of AMPK family kinases, is also shown to function as a class II HDAC kinase in other cell types such as skeletal muscle cells (Berdeaux et al., 2007). These findings may provide explanation for some skeletal dysplasia such as brachydactyly type E, that is caused by haploinsufficiency of *HDAC4* (Williams et al., 2010) or deletion and point mutations of *PTHLH*, the gene coding for PTHRP (Klopocki et al., 2010). Interestingly, the expression of HDAC4 is upregulated in OA cartilage, but barely detectable in normal cartilage. The extent of HDAC4 expression is negatively correlated with severity of OA, and the reduction of HDAC4 level leads to a significant repression of proinflammatory cytokine-induced upregulation of matrix-degrading enzymes including MMP13 (Lu et al., 2014). The role of HDAC4 varies at different stages of chondrocyte, and there are other factors involved in HDAC4 regulation of chondrocyte fate, which deserves further investigation.

Elevated HDAC7 expression in human OA may contribute to cartilage degradation through promotion of *MMP13* gene expression (Higashiyama et al., 2010). HDAC7 suppresses proliferation and chondrogenic differentiation of early chondrocytes through impairment of β-catenin activity (Bradley et al., 2015b). However, whether HADC7 affects chondrocyte fate through its histone modifying activity remains to be clarified. Animal studies have shown that endochondral ossification is impaired when HDAC3, 4, 5, and 7 are knocked out (Rice et al., 2020), indicating the involvement of these HDACs in chondrocyte differentiation and maturation during endochondral bone formation.

Loss-of-function *HDAC8* mutations cause Cornelia de Lange syndrome (CdLS), characterized by intellectual disability, well-defined facial features, upper limb anomalies and atypical growth, etc. *HDAC8* mutation leads to increased acetylation of structural maintenance of chromosomes protein 3 (SMC3), a subunit of the cohesion complex, and inefficient dissolution of the ‘used’ cohesin complex released from chromatin in prophase and anaphase (Deardorff et al., 2012). This result indicates that HDAC8 functions as a vertebrate deacetylase of SMC3. Classic CdLS can be easily diagnosed from birth because of distinctive craniofacial appearance and growth pattern and limb malformations, while other patients show variant clinical features characterized by different degrees of facial and limb involvement (Kline et al., 2018). Interestingly, heterozygous *HDAC8* frameshift mutation is shown to co-exist with *SHOX* haploinsufficiency that cause more complexed clinical features (Severi et al., 2019). It is also shown that HDAC8 and HDAC2 are expressed in hypertrophic chondrocytes and knockdown of HDAC2/3/8 increases *SOX9* and decreased *RUNX2* expression (Chen W. et al., 2016).

Mice lacking *Hdac9* and its truncated variant, HDAC-related protein (HDRP), exhibit polydactyly in their hind limbs in association with overactivity of Sonic Hedgehog (SHH) signaling (Morrison and D’Mello, 2008). This phenotype suggests that HDAC9/HDRP plays a role in the regulation of limb patterning. Noticeably, HDAC9/HDRP induced deacetylation of Nkx3.2 triggers protein inhibitor of activated STAT protein gamma (PIASy)-mediated SUMOylation, which subsequently promotes RING finger protein 4 (RNF4)-mediated SUMO-targeted ubiquitination. This process is involved in the regulation of chondrocyte survival and hypertrophic maturation during cartilage development (Choi et al., 2016).

SIRT1 is shown to be involved in the regulation of *COL2A1* conducted by GCN5 and p300, and functions by associating with other transcription factors. SIRT1 deacetylates and thereby activates peroxisome proliferator-activated receptor gamma coactivator 1alpha (PGC1α). The association of SIRT1 with PGC1α and SOX9 and the subsequent deacetylation of both proteins may aid in the activation of PGC1α and the recruitment of GCN5 and p300, leading to the enhancement of chromatin activity, as indicated by upregulation of H3K9/K14ac and H4K5ac in the promoter of *COL2A1* gene (Dvir-Ginzberg et al., 2008). This suggests that SIRT1 may coordinate with HATs to modulate histone acetylation states in the promoter of *COL2A1* gene, which subsequently regulate chondrocyte function. A recent study indicates that SIRT1 significantly upregulates the expression of *SOX9*, *COL2A1*, and *ACAN* in BMP2-induced chondrogenic differentiation of MSCs, and reduces apoptosis and decomposition of ECM under oxidative stress (Lu et al., 2020). These results indicate that SIRT1 exerts synergistic effect on BMP2-induced chondrogenesis of MSCs and plays an important role in blocking oxidative stress to create a more suitable microenvironment for chondrogenesis of MSCs. In addition, SIRT1 is downregulated in human aged and OA cartilage, and is shown to maintain autophagy in chondrocytes. However, in this case, the deacetylation site of SIRT1 is not on histones but directly on crucial autophagy proteins, including Beclin1, ATG5, ATG7 and LC3 (Fujita et al., 2011; Sacitharan et al., 2020). Similarly, deacetylation by SIRT3 is observed to be involved in the regulation of acetylation of superoxide dismutase 2 (SOD2) in the mitochondria. In the aged mice, SIRT3 is substantially decreased in articular chondrocytes accompanied by decreased SOD2 activity due to its elevated post-translational lysine acetylation, consequently leading to OA. In human OA cartilage, SOD2 is also acetylated, while its activity could be increased with SIRT3 treatment (Fu et al., 2016). These results indicate that restoration of SIRT3 may serve as a therapy to protect articular cartilage from oxidative stress by rescuing acetylation-dependent inhibition of SOD2 activity. SIRT6 is also significantly decreased in articular chondrocytes of OA patients compared with that of normal human. Overexpression of *Sirt6* suppresses chondrocyte replicative senescence by downregulation of NF-κB dependent genes. In mouse OA model, intra-articular injection of lentiviruses containing *Sirt6* gene protects chondrocytes from degeneration (Wu et al., 2015). This suggest that SIRT6 activators may have therapeutic potential for the treatment of OA by reducing inflammatory responses and chondrocyte senescence.

Although originally identified as enzymes that deacetylate histones, a growing number of non-histone substrates have been identified for HDACs, suggesting their general role in regulating protein functions. Elucidating whether an HDAC regulates gene transcription through modification of histones or non-histone proteins is critical in selection of HDAC as a therapeutic target by defining the treatment modality and elimination of potential side effects.

### Histone Lysine Demethylases (KDMs)

The enzymes that remove the histone methyl groups are termed KDMs. Histone lysine (K)-specific demethylase 1 (LSD1, also known as KDM1A) is a flavin-containing amino oxidase that specifically catalyzes the demethylation of mono- and di-methylated histone 3 lysine 4 (H3K4) residues and acts as a transcriptional repressor (Shi et al., 2004). Multiple KDM members have been shown as important regulators involved in the regulation of chondrocyte differentiation and the pathogenesis of OA (Table 2).

The level of LSD1 is elevated in OA compared with that of normal cartilage (El Mansouri et al., 2014). In mice older than 6 months, the increased LSD1 recruitment to the *Sox9* promoter downregulates the level of H3K4me2, which leads to suppression of *Sox9* expression (Zhang et al., 2016b). The transcription of *Nfat1* gene in aged mouse articular chondrocytes is negatively regulated by *Lsd1* through demethylation of H3K4me2 at its promoter region (Zhang et al., 2016a). NFAT1 is a transcriptional regulator of several anabolic and catabolic genes in articular cartilage, such as *COL2A1*, *ACAN*, and *MMP13* (Rodova et al., 2011). KDM2A also catalyzes demethylation of H3K4, as evidenced by the observation that silencing of KDM2A elevates expression of *SOX2* and *NANOG* and enhances chondrogenic differentiation potential in human stem cells from the apical papilla of teeth through upregulation of H3K4me3 at the promoters of these genes (Dong et al., 2013). It is also found that LSD1 promotes transcription of target genes by ligand-induced demethylation of mono- and di-methylated H3K9 (Perillo et al., 2008). Interleukin (IL)-1β induces microsomal prostaglandin E synthase 1 (*mPGES-1*) expression via recruitment of LSD1, which decreases mono- and di-methylated H3K9 level at the *mPGES-1* promoter and is elevated in OA compared with normal cartilage (El Mansouri et al., 2014). mPGES-1 catalyzes the terminal step in the biosynthesis of PGE_2_, a key mediator of OA pathogenesis (Miwa et al., 2000). KDM4B, also known as Jumonji C domain-containing 2B (JMJD2B), selectively reduces H3K9me3 marks to H3K9me1 with the H3K9me2 state remaining unaltered (Klose et al., 2006; Whetstine et al., 2006). During TGF-β-mediated chondrogenesis of human MSCs, KDM4B removes the repressive epigenetic mark, H3K9me3, from the *SOX9* gene promoter region and activates the transcription of *SOX9* through SMAD3 binding in the *SOX9* gene promoter (Lee et al., 2016). These results indicate that KDM4B epigenetically regulates chondrogenesis *in vitro*, while its involvement in cartilage development and pathogenesis of OA remains lacking.

H3K27 methylation is removed by the demethylases, ubiquitously transcribed X-chromosome tetratricopeptide repeat protein (UTX, also known as KDM6A) and Jumonji domain containing-3 (JMJD3, also known as KDM6B) (Swigut and Wysocka, 2007). UTX decreases H3K27me3 level, and upregulates the expression of *SOX9*, *COL2A1*, and *ACAN*, resulting in chondrogenic differentiation of periodontal ligament stem cells (PDLSCs) (Wang et al., 2018a). JMJD3 is shown to be elevated during chondrogenic differentiation of murine MSCs, leading to a downregulation of H3K27me3. The binding site of JMJD3 is presumed to be the promoters of *COL2A1* and *ACAN* genes (Dai et al., 2017). Consistently, *Jmjd3*-deficient mice exhibit markedly reduced proliferation and hypertrophy of chondrocytes, as well as a severe delay of endochondral ossification. JMJD3 interacts with and enhances RUNX2 activity, and potentiates *Runx2* and *Ihh* transcription as a major eraser of H3K27me3 at the promoters of *Runx2* and *Ihh* genes during chondrocyte maturation (Zhang F. et al., 2015). Notably, it is JMJD3 but not UTX expression that is increased during MSC chondrogenesis and in damaged OA cartilage, suggesting a predominant role of JMJD3 in chondrogenesis and OA development (Huang et al., 2020). In addition, AT-rich interactive domain 5b (Arid5b) serves as a transcriptional coregulator of SOX9 to recruit PHD finger protein 2 (PHF2), the H3K9me2 demethylase, to the promoters of *Col2A1* and *Acan* genes, subsequently promotes chondrogenic differentiation. Mice lacking Arid5b exhibit growth retardation with delayed endochondral ossification (Hata et al., 2013). This data suggests that PHF2 functions as an important KDM to mediate Arid5b/SOX9 controlled chondrogenesis.

## Readers Regulating Chondrocyte Function

Taking advantage of the traditional pull-down method, the structure-function based educated guess, and protein array or chemical biology based high through-put screening approaches, a large panel of histone readers have been identified. Multiple excellent reviews have systematically summarized histone readers and histone combinatorial readout associated with PTMs (Yun et al., 2011; Wang et al., 2015; Andrews et al., 2016; Hyun et al., 2017; Zhao et al., 2019). Epigenetic reader proteins that recognize and bind histone marks provide a crucial link between histone modifications and their biological functions. A panel of acetylation and methylation readers have been identified for their involvement in the regulation of chondrocyte differentiation and function, including bromodomain (BRD), PHD fingers, WD40 domains, and ankyrin repeats (Table 3).

### Histone Acetylation Readers

Bromodomain is the first histone modification reader identified to bind specifically acetyl-lysine in peptides from histones H3 and H4 (Dhalluin et al., 1999). BRD and extra terminal domain (BET) family proteins include BRD2, BRD3, BRD4 and BRD testis-specific (BRDT). These proteins recognize the acetylated lysine residues in histones H3 and H4 and alter chromatin structure (Zeng and Zhou, 2002; Marmorstein and Zhou, 2014; Shi and Vakoc, 2014). Among them, BRD4 is the most widely studied. BRD4 plays an essential role in activating the positive transcription elongation factor b (P-TEFb), a cyclin dependent kinase that is composed of cyclin dependent kinase Cdk9 and cyclin T (CycT) (Jang et al., 2005; Yang et al., 2005). BRD4 stimulates P-TEFb kinase activity for phosphorylation of the C-terminal domain (CTD) of RNAP II (Itzen et al., 2014). As a reader, BRD4 also associates with p300/CBP and enhances H3ac. In ESCs, the region that co-associates with BRD4 and p300 has a greater abundance of H3K27ac and H3K56ac than the region occupied by p300 alone, suggesting that BRD4 facilitates p300 and CBP HAT activities on cell-specific enhancers and promoters. Interestingly, BRD4-p300 participates in the regulation of mesoderm formation (Wu et al., 2018). In addition, in the absence of p300, there is an intrinsic HAT activity at the C-terminal of BRD4. BRD4 acetylates H3K122, a residue critical for nucleosome stability, resulting in nucleosome eviction and chromatin decompaction (Devaiah et al., 2016). Increasing evidence shows that BRD4 is an important reader protein involved in regulating chondrocyte functions.

BRD4 can promote gene transcription in two ways. The first is to recruit the positive transcription elongation factor (P-TEF) to the upstream non-promoter region of high mobility group protein B1 (HMGB1) to induce Ser-2-phosphorylation of the carboxy-terminal domain of RNAP II, ultimately enhancing transcriptional elongation of *HMGB1* gene (Zhang et al., 2012; Jiang et al., 2017). HMGB1 plays an important role in regulating inflammation by stimulating production of inducible nitric oxide synthase (iNOS), nitric oxide (NO), IL-1β and IL-8, as well as inducing the production of damage-associated molecular markers of OA, such as MMP3 and MMP13 (Amin and Islam, 2014). The other way is to directly bind acetylated lysine side chains of modified histones. It is noteworthy that histone acetylation is involved in the regulation of IL-1β or TNF-α-induced transcription of matrix-degrading enzyme genes. For example, IL-1β induces H4K5ac at *MMP1*, H4K5ac at *MMP3*, and H4K12ac at *ADAMTS4*, and TNF-(α induces H4K8ac at MMP1 and H4K12ac at MMP3. The increased H4K5/8/12ac in the promoter regions of these genes might be responsible for the recruitment of BRD3 and BRD4, thereby leading to enhancement of gene transcription associated with articular cartilage degeneration (Dai et al., 2019). Notably, BRD4 level is shown to be positively correlated with degeneration of articular cartilage and the activation of inflammation related NF-κB signaling pathway in patients with OA (Jiang et al., 2017). In addition, BRD4 is reported to play an important role in fibrosis through direct binding of H3K27ac in the *COL1A1* gene, which is a major factor in the induction of tissue fibrosis (Ding et al., 2015). During initiation of OA, articular cartilage fibrosis occurs and is accompanied by upregulation of *COL1A1.* Whether or not BRD4 participates in this early stage of pathogenesis of OA remains an interesting question.

### Histone Methylation Readers

Early studies have identified WD repeat domain 5 (WDR5), a member of the WD40 repeat proteins, as a reader of the Set/Ash HAT complex essential for H3K4me3, and knockdown of *Wdr5* in the early embryo of zebrafish causes developmental defects of the somite (Wysocka et al., 2005). This family of proteins have been implicated in numerous cellular functions including signal transduction, mRNA processing, gene regulation, vesicular trafficking and regulation of the cell cycle (Gori et al., 2005). A series of studies show that *Wdr5* mRNA is expressed in bone marrow stromal cells, osteoblasts, osteocytes and chondrocytes, and dramatically accelerates the program of osteoblastic and chondrocyte differentiation *in vitro* (Gori and Demay, 2004; Gori et al., 2006). Mice overexpressing *Wdr5* under the control of the mouse *Col1A1* promoter (*Col1-Wdr5*) exhibit a larger zone of hypertrophic chondrocytes in developing long bones at embryonic day (E) 14.5 (Gori et al., 2006). The mutants also show enhanced chondrocyte differentiation though upregulation of Twist-1 and FGF18 in the perichondrium, which is associated with transcriptional control of *Runx2* by WDR5 (Gori et al., 2009). These findings indicate that WDR functions on both histone methylation and transcriptional regulation of the key transcription factor controlling chondrocyte fate and skeletal development.

The plant homeodomain (PHD) fingers are central readers of PTMs that exist in more than 100 human proteins, many of which bind to unmodified or methylated H3K4. The PHD fingers play crucial roles in regulation of gene expression and carcinogenesis (Tsai et al., 2010; Zeng et al., 2010; Jain et al., 2020). It is shown that tripartite motif-containing protein 24 (TRIM24) functions as a reader of dual histone marks through tandem PHD and BRD domains (Tsai et al., 2010). Knockdown of *Trim24* upregulates receptor-interacting protein kinase-3 (RIP3) and promotes OA pathogenesis in the mouse OA model, suggesting that the TRIM24-RIP3 pathway may serve as a new target for treatment of OA (Jeon et al., 2020). However, the direct link between TRIM24 and PHD domains in chondrocytes deserves to be further elucidated.

Ankyrin repeats are segments of 32–33 amino acids typically found in clusters of four or more contiguous repeats (Brent and Marmorstein, 2008). A recent study shows that p100 mice (p100–/–) with homozygous deletion of the COOH-terminal ankyrin repeats exhibit dwarfism, abnormal arrangement of chondrocyte columns and a narrowed hypertrophic zone in the growth plate, accompanied by the suppression of *Col10A1* and *Mmp13* expression. Furthermore, these defects are partly rescued when the *RelB* gene is deleted in the *p100*–/– mice. These data indicate that the constitutively activated alternative NF-κB pathway suppresses chondrocyte proliferation and differentiation (Nakatomi et al., 2019). That the histone methylation readers regulate chondrocyte fate and OA pathology provides valuable information for exploration of novel therapeutic targets for OA treatment.

## Histone-Modifying Enzymes as Potential Therapeutic Targets for OA

### HMT Inhibitors (HMTi’s) as Potential Therapeutics for OA

Methylthioadenosine (MTA), an HMTi, dose-dependently suppresses IL-1-induced *iNOS* and *COX-2* expression in human chondrocytes accompanied by the decline in H3K4 methylation (El Mansouri et al., 2011). EZH2 level is significantly increased in chondrocytes of articular cartilage in OA patients, and treatment with EZH2 inhibitor EPZ005687 decreases *MMP-13*, *ADAMTS-5* and *COL10A1* expression by inhibition of β-catenin signaling in chondrocytes. Intra-articular delivery of EPZ005687 delays OA development in the mouse OA model (Chen L. et al., 2016). Another EZH2 inhibitor EPZ-6438 is found to reduce IL-1β-induced expression of genes associated with inflammation and pain (e.g., *NO*, *PGE2*, *IL-6*, and *NGF*) and catabolism (e.g., *MMPs*) in chondrocytes. Intra-articular injection of EPZ-6438 reduces cartilage degradation and improves joint motor functions in the mouse OA model (Allas et al., 2020). In addition, EPZ-6438 rescues chondrogenic differentiation potential of periodontal ligament stem cells (PDLSCs) after knockdown of KDM6A by regulating H3K27me3 (Wang et al., 2018a). 3-Deazaneplanocin A (DZNep), an inhibitor of *S*-adenosyl-L-homocysteine hydrolase (SAHH) known to inhibit EZH2, counteracts IL-1β-induced expression of genes involved in cartilage matrix breakdown (e.g., *MMPs* and *ADAMTS*), while upregulating *SOX9* and *COL2A1* expression (Aury-Landas et al., 2017). H3K9 methylation level is reduced in the degenerating condylar articular cartilage during development of OA in the temporomandibular joint. Chaetocin, a selective H3K9 methylation inhibitor, increases *Mmp1* and *Mmp13* while suppresses *Sox9* and *Col2A1* mRNA levels in ATDC5 mouse chondroprogenitor cells (Ukita et al., 2020). These results suggest that HMTi’s may serve as potential therapeutic agents to protect articular cartilage from degeneration through inhibition of inflammatory responses and catabolic metabolism of chondrocytes during OA.

### Histone Demethylase Inhibitors as Potential Therapeutics for OA

GSK-J4, an inhibitor of histone demethylases JMJD3 and KDM6A (UTX), suppresses IL-1β-induced production of pro-inflammatory cytokines (IL-6 and IL-8) and catabolic enzymes (MMP-9 and ADAMTS-5), as well as inhibits degradation of collagen type II and aggrecan. *In vivo*, GSK-J4 prevents articular cartilage from degeneration in the destabilization of the medial meniscus (DMM) induced mouse OA model (Zhou et al., 2020). GSK-J4 is also found to prevent *ex vivo* cartilage destruction and inhibit the expression of OA-related genes such as *MMP13* and *COX-2*. In an engineered chondrogenesis model using human MSCs, GSK-J4 inhibits *SOX9* and *COL2A1* expression and chondrocyte ECM production (Yapp et al., 2016). This suggests that targeted inhibition of H3K27me3 demethylases might provide a novel approach for OA therapy, while enhancing its activity might improve production of engineered cartilage formation. Detailed molecular mechanisms underlying these distinct functions of GSK-J4 remain to be defined.

Pargyline and tranylcypromine, the inhibitors of LSD1, are shown to prevent IL-1β-induced H3K9 demethylation at the microsomal prostaglandin E synthase 1 (*mPGES-1*) promoter and inhibit the expression of *mPGES-1* in human OA chondrocytes (El Mansouri et al., 2014). These findings may have implications in the identification of novel anti-inflammatory drugs for OA therapy.

Stem cell-based therapy is an attractive therapeutic approach for the treatment of a variety of degenerative diseases, including OA. A recent study shows that synovial MSC (SMSC)-derived, extracellular vesicle (EV)-packaged miR-31 potentiates chondrocyte proliferation and migration as well as cartilage formation by targeting KDM2A, which binds to the transcription factor E2F1 and inhibits its transcriptional activity. SMSC-EVs and EVs from miR-31-overexpressed SMSCs alleviates cartilage damage and inflammation in a mouse OA model (Wang et al., 2020). These findings indicate that microRNA based epigenetic regulation and histone demethylation may interact to regulate inflammatory responses and chondrocyte ECM production during OA, and provide a novel approach for the design of effective OA therapeutics.

### HDAC Inhibitors (HDACi’s) as Potential Therapeutics for OA

HDACi’s include a panel of chemical compounds and natural product derived small molecule compounds function to inhibit HDACs and have been applied for treatment of multiple diseases (Bassett and Barnett, 2014; Khan and Haqqi, 2018). Several chemical compound HDACi’s have been approved by the United States Food and Drug Administration (FDA) for treatment of cancers (Vorinostat, Romidepsin) and mental disorder (Valproic acid, Valproate, Divalproex sodium, Depakote) (Suraweera et al., 2018). Some other HDACi’s, such as Panobinostat (LBH589), Entinostat (MS-275), and Givinostat (ITF2357), are currently under evaluation in clinical trials for treatment of inflammation, neurodegeneration or diabetes (Dinarello et al., 2011; Culley et al., 2013). HDACi’s are also considered as important candidates for the development of efficient approaches for stem cell- or tissue engineering-based regenerative therapies (Lawlor and Yang, 2019). The HDACi, Givinostat (ITF2357), shows positive effects on inhibiting arthritic phenotypes in the systemic-onset juvenile idiopathic arthritis (Vojinovic et al., 2011). Up to date, no HDACi has been approved for the treatment of OA, but increasing evidence suggests that HDACi’s possess potential protective effects on articular cartilage.

Trichostatin A (TSA), a pan-HDACi, isolated from *Streptomyces hygroscopicus*, has been tested in a variety of cellular or explant culture models and OA animal models. TSA and sodium butyrate potently inhibit cartilage degradation in a bovine nasal cartilage explant assay. Both HDACi’s may serve as chondroprotective agents through the inhibition of the expression of destructive metalloproteinases (e.g., MMP-1 and MMP-13) and aggrecan-degrading enzymes (e.g., ADAMTS4, ADAMTS5, and ADAMTS9) by chondrocytes (Young et al., 2005). However, some controversial results indicate that TSA has no effect on *MMP1* expression, despite an increase in H3 acetylation at the *MMP1* gene promoter (Clark et al., 2007). In addition, TSA acetylates histone H3K9/14 at the *leptin* promoter and increases *leptin* expression, subsequently upregulates *MMP13* expression. In normal chondrocytes, *leptin* is minimally expressed, and the expression of *MMP13* is suppressed (Iliopoulos et al., 2007). This characteristic suggests that *leptin* may serve as a potential therapeutic target for OA through suppression of the acetylation state of its promoter. In a rabbit OA model, TSA prevents articular cartilage from degradation and inhibits production of MMPs (MMP-1, MMP-3, and MMP-13), cathepsins (K, B, L), and IL-1 (Chen et al., 2011). Systemic administration of TSA also prevents articular cartilage destruction in a mouse OA model (Young et al., 2005). Interestingly, TSA does not show significant protection of articular cartilage degeneration in the OA model in the nuclear factor (erythroid-derived 2)-like 2 (*Nrf2*) knockout mice, suggesting that *Nrf2* is required for TSA-dependent protective effect in OA (Cai et al., 2015). TSA also suppresses the mechanical stress or IL-1β induced expression of *RUNX2* and *ADAMTS5* by inhibiting the activation of MAPK signaling (ERK1/2, p38MAPK, JNK) in human chondrocytes (Saito et al., 2013; Wang et al., 2018b). In IL-1β-treated primary human chondrocytes, TSA upregulates *ACAN* expression and inhibits *MMP13* expression (Meng et al., 2018). Vorinostat inhibits production of various MMPs and NO induced by IL-1β in human chondrocytes through inhibition of p38 and ERK1/2 signals (Zhong et al., 2013). Vorinostat and Panobinostat markedly increase miR-146a expression in OA fibroblast-like synoviocytes (OA-FLS), and inhibit IL-1β-induced IKK/IκB/p65 phosphorylation and IL-6 secretion, suggesting the potential rationale of anti-inflammatory effects associated with OA (Wang et al., 2013). It is further shown that Vorinostat is an effective suppressor of IL-6 induced signaling events in OA (Makki and Haqqi, 2016). As mentioned above, miR-193b-3p inhibits HDAC3 expression, and promotes H3 acetylation in the *SOX9*, *COL2A1*, *ACAN*, and *COMP* gene promoters (Meng et al., 2018), suggesting that specific miRNAs may also serve as HDACi’s to maintain chondrocyte phenotype. These studies strongly suggest that HDACi’s are potential agents for the management of OA through selective inhibition of molecular targets associated with inflammatory responses and articular cartilage degeneration during OA.

### Reader Inhibitors as Potential Therapeutics for OA

p300 and CBP are large, multi-domain proteins, which in addition to their HAT domain, possess bromodomains that bind to acetylated histones especially H3K27ac and H3K56ac, and are required for chromatin binding (Picaud et al., 2015; Ebrahimi et al., 2019). As indicated above, HATis and HDACi’s (e.g., TSA) possess very broad effects on protein modifications, while BET inhibitors (e.g., JQ1) have more narrowly defined effects. CBP30, a specific p300/CBP inhibitor, possesses an even more restricted effect on gene expression than that observed with the pan-BET inhibitor JQ1. CBP30 marks molecular specificity for the bromodomains of p300/CBP (Hammitzsch et al., 2015). This selective targeting effect of CBP30 might potentially lead to fewer side effects than the broadly acting epigenetic inhibitors (Hammitzsch et al., 2015). CBP30 has been shown to enhance induced pluripotent stem cell (iPSC) reprogramming, indicated by robust expression of the pluripotency markers, OCT4, SOX2, NANOG, and SSEA4 (Ebrahimi et al., 2019). This activity suggests that CBP30 may serve as an agent to facilitate iPSC reprogramming and stem cell expansion. It is thus worthwhile to explore if CBP30 also functions in the expansion of chondroprogenitors. As a BRD4 antagonist, JQ1 is shown to reduce inflammatory and catabolic pathways in chondrocytes, and attenuates cartilage destruction in mice with destabilized joints induced by anterior cruciate ligament transection (Jiang et al., 2017). The pan-BET inhibitors, I-BET151 and JQ1, have inhibitory actions on chondrocyte metabolism and fate control. On the one hand, the expression of catabolic genes including *iNOS*, *COX2*, *RUNX2*, *IL8*, *IL1B*, *MMP2*, *MMP9*, *MMP13*, and *ADAMTS5* is suppressed. On the other hand, the expression of chondrogenic mark genes *SOX9*, *ACAN* and *COL2A1* is also downregulated (Dai et al., 2018; De-Andrés et al., 2018). The BET inhibitors may represent a category of potential candidates that deserve to be tested in chondrocytes and OA animal models.

Histone-modifying proteins that are involved in the pathogenesis and progression of OA are illustrated in Figure 2. And inhibitors of histone-modifying enzymes that have been tested in the OA animal models and might serve as potential therapeutics for OA are summarized in Table 4.

**TABLE 4:** Inhibitors of histone-modifying enzymes as potential therapeutics for OA treatment.

Molecule/drug	Enzyme inhibitor	Gene regulation	Species	Effects on chondrocyte or cartilage	References
EPZ-6438	EZH2i	*Mmp1, Mmp3 Mmp13*↓*; Ngf*↓	Murine	Reducing cartilage degradation, preserving proteoglycan	Allas et al., 2020
EPZ005687	EZH2i	*Mmp13, Adamts5* and *Col10A1*↓	Murine	Delayed OA development in mice model	Chen L. et al., 2016
3-Deazaneplanocin A (DZNep)	SAHHi	*NO, PGE2, MMP*↓ *COL2A1, SOX9*↓	Human	Anti-inflammatory and chondroprotective effects	Aury-Landas et al., 2017
Trichostatin A (TSA)	HDACi	*Mmp1, Mmp3, Mmp13*↓ *Tnf-α, II-1β, II-6*↓	Murine	Reduced cartilage damage in mouse OA model	Cai et al., 2015
	HDACi	Cathepsins K, B, L, S, and cystatin C↓,	Rabbit	Protective effects against cartilage degradation in rabbit OA model	Chen et al., 2011
	HDACi	Cytokine-induced *MMP1, MMP13*↓	Human, murine	Repressing MMPs expression, inhibition of cartilage resorption	Culley et al., 2013
	Pan-HDACi	*Leptin, MMP13*↑	Human	Increasing leptin expression	Iliopoulos et al., 2007
	Pan-HDACi	*RUNX2, ADAMTS5*↓	Human	Inhibiting MAPK signaling (ERK1/2, p38MAPK, JNK)	Saito et al., 2013
MS-275	HDACi	Cytokine-induced MMP1, MMP13↓	Human murine	Repressing cytokine-induced metalloproteinase expression in cartilage cells, resulting in inhibition of cartilage resorption	Culley et al., 2013
Vorinostat	HDACi	*MMP1, MMP13*↓ *iNOS*↓*; TIMP-1*↑	Human	Inhibiting NF-κB pathway in chondrocytes	Zhong et al., 2013
Panobinostat	HDACi	miR-146a ↑	Human	Suppressing IKK/IκB/p65 phosphorylation, and IL-6 secretion	Wang et al., 2013
Chaetocin	HMTi	*Mmp1, Mmp13↑ Sox9, Col2A*1↓	Murine	Promoting the progress of OA	Ukita et al., 2020
GSK-J4	KDM6Ai	*Mmp9, Adamts5*↓*; II-6, II-8*↓	Murine	Inhibiting degradation of Collagen type II and aggrecan	Zhou et al., 2020
Pargyline and tranylcypromine	LSD1i	*mPGES-1*↓	Human	Inhibiting *mPGES-1* in human OA chondrocytes	El Mansouri et al., 2014
JQ1	BRD4i	*iNOS, COX2, IL8, IL1B, MMP13*↓	Human	Inhibiting cartilage degradation	De-Andrés et al., 2018
I-BET151	BETi	*MMP1, MMP3,MMP13I*↓ *ACAN,COL2A1,SOX9*↓	Murine human	Inhibiting inflammatory factors in chondrocytes	Dai et al., 2018

## Conclusion and Future Perspectives

In this review, we have presented an overview of the advances in the understanding of the molecular control of histone modification proteins, particularly the writers, erasers and readers, involved in the regulation of chondrocyte fate, cartilage development and a variety of chondropathies. The findings clearly indicate a complex and sophisticated control of histone modifications in the regulation of downstream signaling molecules that control chondrocyte gene expression programs. We have highlighted recent progress on the use of small molecules or drugs to manipulate histone signals to regulate chondrocyte functions or treat cartilage lesions, particularly OA, in pre-clinical animal models, and discussed their potential therapeutic benefits and limitations for preventing articular cartilage degeneration or promoting its repair or regeneration.

Most of the above mentioned histone-modifying proteins are able to modify multiple lysine residues on histones functionally associated with different genes in chondrocytes (Tables 1–3 and Figures 1, 2). It is noteworthy that the targets of these histone-modifying proteins seem to be relatively non-specific. The molecular writers and erasers can also modify non-histone proteins, such as nuclear receptors and transcription factors, to modulate chondrogenic gene expression. As multiple histone modification proteins may cross-talk to participate in the regulation of chondrogenic gene programs, pharmacological manipulation of these histone-modifying proteins may cause a variety of responses within chondrocytes or even lead to potential off-target side effects to non-cartilage tissues.

It remains challenging to define the precise, gene-specific histone modifications in chondrocyte fate determination. There are several possible directions for future studies. (1) A more comprehensive understanding of histone modifications will help to unravel how histone modifying enzymes are specialized to achieve epigenetic control. A comprehensive study on the interactions of transcription factors and histone-modifying enzymes, or “histone code,” is needed. (2) It is worthwhile to clarify the different roles of histone modifications at different developmental stages of chondrocytes, because a histone modification that inhibits the early stage of chondrogenesis may have the potential to benefit chondrocyte function in later stages of development. Thus, selection of therapeutic molecules or drugs must be adjusted to target specific developmental states of the cartilage. (3) Drug combinations that target histone modifying enzymes should also be explored in order to achieve better efficacy of treatment. (4) Discovery of small molecules that can precisely target specific histone modifying proteins is a promising approach for the treatment of cartilage lesions. (5) With the development of gene-editing techniques, including helper-dependent adenoviral vector, traditional meganucleases, ZFNs, TALENs, and CRISPR/Cas9, manipulation of the genetic material at specific locations in an organism’s genome has become feasible (Gaj et al., 2013; Chen et al., 2015; Munsell et al., 2018; Gu et al., 2019; Zhang et al., 2020). CRISPR/Cas9 is by far the most convenient gene-editing tool for basic and clinical studies (Chen et al., 2015; Zhang et al., 2020), and has been used for manipulation of epigenetic status at the specific genomic region to investigate the respective therapeutic potentials (Kearns et al., 2015). The CRISPR/Cas9 gene-editing technique holds great promise for the manipulation of histone modifications as a therapy for OA, osteochondrodysplasia, and stem cell- or tissue engineering-based regenerative therapies.

Taken together, histone modifications indisputably play essential roles in the modulation of the biological behavior of chondrocytes. Precise manipulation of these modulating effects in chondrocytes will not only facilitate the understanding of the functions of histone modifications, but also the discovery of novel therapeutic approaches for OA, osteochondrodysplasia, and articular cartilage repair or regeneration.

## Author Contributions

RT and CW conceived the concept and wrote the manuscript. All authors listed have made a substantial contribution to collecting and analyzing data, revising, as well as approving the revised manuscript.

## Conflict of Interest

The authors declare that the research was conducted in the absence of any commercial or financial relationships that could be construed as a potential conflict of interest.
